# Emergency Clinicians’ Perceptions of Communication Tools to Establish the Mental Baseline of Older Adults: A Qualitative Study

**DOI:** 10.7759/cureus.20616

**Published:** 2021-12-22

**Authors:** Anita Chary, Christopher Joshi, Noelle Castilla-Ojo, Ilianna Santangelo, Kei Ouchi, Aanand D Naik, Christopher R Carpenter, Shan W Liu, Maura Kennedy

**Affiliations:** 1 Emergency Medicine, Massachusetts General Hospital, Boston, USA; 2 Medicine, University of Texas Southwestern Medical Center, Dallas, USA; 3 Graduate Education, Harvard Medical School, Boston, USA; 4 Emergency Medicine, Brigham and Women's Hospital, Boston, USA; 5 Psychosocial Oncology and Palliative Care, Dana-Farber Cancer Institute, Boston, USA; 6 Houston Center for Innovations in Quality, Effectiveness and Safety, Michael E. DeBakey Veterans Affairs (VA) Medical Center, Houston, USA; 7 Internal Medicine, Baylor College of Medicine, Houston, USA; 8 Emergency Medicine, Barnes Jewish Hospital, St. Louis, USA; 9 Emergency Care, Washington University School of Medicine, St. Louis, USA; 10 Emergency Medicine, Harvard Medical School, Boston, USA

**Keywords:** emergency medicine, geriatrics, delirium, altered mental status, communication tools

## Abstract

Background

Evaluating older adults with altered mental status in emergency settings can be challenging due to the inability to obtain a history from patients directly and limited collateral information about the change from a patient’s mental status baseline. Documents and videos establishing a patient’s mental baseline could represent useful communication tools to aid emergency clinicians.

Methods

Qualitative interviews conducted with 22 emergency clinicians (12 physicians and 10 advanced practice providers) identified methods they use to determine baseline mental status of older adults in the ED and the perceived utility of document- and video-based information about an older adult’s baseline mental status. Interview transcripts were coded for dominant themes using deductive and inductive approaches.

Results

Participants determine an older adult’s baseline mental status by obtaining information about the patient’s baseline cognition (memory and communication) and function (activities of daily living and mobility). The techniques they use include 1) reviewing the electronic medical record, 2) speaking with family members or caregivers by phone or in person, and 3) obtaining verbal or phone reports from emergency medical services personnel or health care providers from short- or long-term care facilities. The majority of participants thought that a document or video with information about a patient’s baseline mental status would be useful (n=15, 68%), qualifying that content ought to be brief, clearly dated, and periodically updated.

Conclusions

Documents or videos could assist emergency clinicians in establishing baseline cognitive function when evaluating geriatric patients and may have implications for improving the detection of delirium.

## Introduction

Altered mental status accounts for approximately 25% of emergency department (ED) visits among older adults [1]. Clinicians use the broad term “altered mental status” to refer to changes in cognition, level of consciousness, and behavior, which may result from diagnoses such as delirium, mild cognitive impairment, and dementia [2-4]. Delirium, a syndrome of disturbance in consciousness and attention which may be provoked by acute illness, is undetected in at least two-thirds of ED cases [1,5,6]. When detected in the ED, delirium is associated with prolonged hospitalization, falls, and increased mortality [7-9]. Cognitive impairment, or difficulty with memory and executive functioning, is similarly underrecognized in EDs [1,10].

ED evaluation of older adults with altered mental status can be challenging, as emergency clinicians cannot always obtain an accurate history from patients themselves [7,11,12]. Information from family, caregivers, or residential care professionals about a patient’s baseline mental status and any changes from it (i.e., collateral information) is often missing or not readily available on presentation to the ED [13]. These challenges have been amplified by the COVID-19 pandemic with infection control measures of social distancing and hospital visitor restrictions [14]. Lack of collateral information can lead to delays in establishing diagnoses and under-recognition of delirium [7], which itself can be a presenting feature of COVID-19 among older adults [15].

Communication tools, or instruments that convey information about patients and serve as adjuncts to history-taking and chart review, could improve clinical evaluation of patients who cannot provide their own history. As one example, the Alzheimer’s Society developed a written packet entitled “This is me” to characterize the baseline cognition and function of a person with dementia for healthcare providers and professional caregivers in home, hospital, and residential care settings [16]. As another example, video-based testimonials communicating older adults’ pre-recorded advanced care plans can help clinicians understand patients’ goals of care and clarify written advanced care planning documentation in times of serious illness [17,18]. Similar communication tools could be deployed to offer insights into an older adult’s baseline cognitive and functional status compared to ED presentation. To our knowledge, such tools do not exist for the purpose of establishing older adults’ baseline mental status. We conducted an exploratory qualitative study with emergency clinicians to assess the feasibility and inform the development of such document- and video-based communication tools, which could ultimately facilitate delirium recognition. Improving emergency clinicians’ ability to evaluate changes in mental status could increase detection of delirium, with potential downstream effects on preventing associated adverse outcomes, both of which are priority areas in geriatric emergency medicine [7,9,11].

## Materials and methods

We performed qualitative interviews with 12 emergency physicians and 10 advanced practice providers regarding the perceived utility of communication tools to demonstrate a patient’s baseline mental status. From February to May 2021, we recruited interview participants via emails to physician and APP list-servs of EDs of two urban academic hospitals and one affiliated community hospital in the United States Northeast.

A research assistant (IS) trained by an experienced qualitative investigator with doctoral training in anthropology (AC) obtained informed consent and conducted interviews over an online audio-visual platform. Interviews focused on clinicians’ experiences evaluating and obtaining collateral information about older adults with altered mental status, prior to and during the COVID-19 pandemic. The qualitative approach to interviewing was phenomenology, which seeks to characterize phenomena as experienced and lived by individuals within a situation. Participants were asked about: 1) the information they sought to determine a patient’s baseline mental status; 2) how useful they would find a document depicting a patient’s baseline mental status, with the interviewer screen sharing the “This is me” packet as an example [16]; and 3) how useful they would find a video depicting a patient’s baseline mental status and a neurological exam.

De-identified transcripts of audio recordings served as the basis for data analysis. A team of six researchers performed thematic analysis using both a deductive approach, in which codes are created based on interview questions, and an inductive approach, wherein themes emerge from data rather than a priori hypotheses. Specifically, the researchers reviewed the first seven transcripts for codes, or repeated patterns and meanings, developed a codebook, had two team members independently apply codes to each transcript, and had a third team member reconcile discrepancies in coding [19]. The researchers elucidated common themes for each code. No new themes arose after 18 interviews, which was confirmed with four additional interviews, indicating data saturation and adequate sample size [20], and study enrollment was concluded. This research was deemed exempt by the Partners Healthcare Institutional Review Board, Boston, MA (Protocol 2020P003925). The description of this investigation follows the Standards for Reporting Qualitative Research guidelines [21].

## Results

Interviews lasted approximately 15 minutes. Participants included eight attending physicians, four resident physicians, eight physician assistants, and two nurse practitioners whose practice spanned two academic hospitals and one community hospital. Half practiced in an academic hospital and half practiced in both academic and community hospitals. Additional demographics are outlined in Table 1. Information about participants’ racial/ethnic identity, gender identity, and age is not reported to avoid the identification of participants. The number of years of emergency medicine clinical practice, rather than age, is reported as a reflection of clinical experience. Themes emerged regarding 1) prioritizing domains of cognition and function when establishing baseline mental status; 2) the perceived utility of a document or video in saving time and improving understanding of a patient’s baseline; and 3) key concerns and suggestions surrounding brevity and updating of information.

**Table 1:** Demographics of Clinician Participants in a Qualitative Study of Emergency Clinicians’ Perceptions of Communication Tools to Establish the Mental Baseline of Older Adults

Characteristic	n (%)
Practice Setting	
Academic	11 (50%)
Academic & Community	11 (50%)
Professional Role	
Attending Physician	8 (36%)
Resident Physician	4 (18%)
Physician Assistant	8 (36%)
Nurse Practitioner	2 (9%)
Years Practicing Emergency Medicine (Range)	
Attending Physician	6 to 41
Resident Physician	1 to 4
Physician Assistant	2 to 17
Nurse Practitioner	1 to 10

Establishing baseline mental status

**Figure 1:**
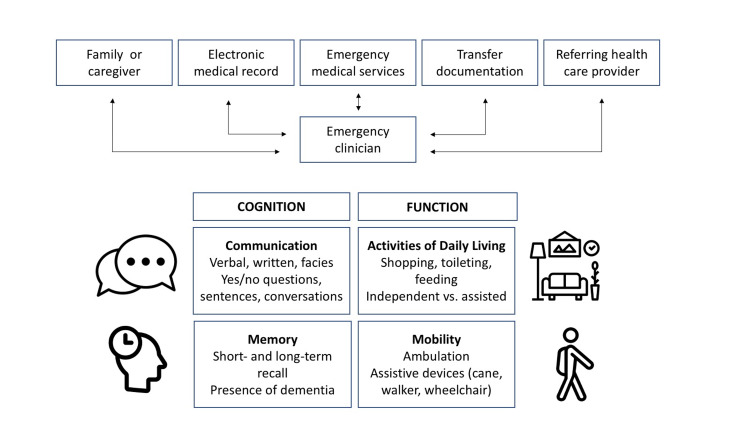
Sources of collateral information and information sought to establish baseline mental status

Participants used multiple sources to obtain information about a patient’s baseline mental status, including the electronic medical record (EMR), remote or in-person conversations with family members and caregivers, reports from emergency medical services personnel, and for patients arriving from short- or long-term care facilities, transfer documentation or verbal reports by phone from referring health care providers. When asked what information they sought to determine an older adult’s baseline mental status, participants’ responses fell under four major categories related to both cognitive and functional status (Figure 1).

1) Communication: The patient’s usual method of communication (verbal, written, facial expressions) and types of information able to communicate (yes/no questions, simple sentences, conversations)

2) Memory: The patient’s short and long-term recall and presence of dementia

3) Activities of daily living (ADLs): The patient’s performance of specific ADLs (shopping, toileting/bathing, feeding) independently vs. with assistance

4) Mobility: The patient’s ability to ambulate and use assistive devices (cane, walker, wheelchair).

Perceived utility of a document

The majority of participants felt that a document with information about a patient’s baseline mental status would be useful (n=15, 68%). The “This is me” packet, which the interviewer shared with participants, contains three pages of written information organized into multiple sections [16]. The content that participants identified as most valuable for emergency clinicians were related to communication, memory, mobility, and ability to perform ADLs, reflecting the categories they prioritized when trying to obtain information about a patient’s mental status baseline. Participants felt that other information described in the “This is me” packet, such as cultural and religious affiliation, habits, triggers of anxiety, and calming measures would be more useful in the inpatient setting, though three participants voiced that such information could help with delirium prevention and management in the ED. Three participants also pointed out that as a whole, the document could help humanize an older adult by helping providers “better understand who they are as a person and as a patient” (Participant 117, attending physician). A minority of participants thought a document would not be useful (n=3, 14%), given emergency clinicians’ time constraints, with two reflecting that a document like “This is me” would be “too long to be useful in the ED” (Participant 119, attending physician). Others qualified that they would find a document useful (n=4, 18%) only if brief and inclusive of key information that could be rapidly reviewed.

Perceived utility of a video

Most participants expressed that they would find a video depicting a patient’s baseline mental status and neurological exam useful (n=15, 68%). Participants noted that EMRs did not always contain accurate or complete information about prior neurologic exams and that they could not rely on family members or caregivers to describe a patient’s prior neurologic exam. Some suggested that capturing baseline speech and mobility on video would allow for rapid and objective comparison to a current ED presentation. For example, as one participant stated: “To the extent that the medical records can't be trusted, having an actual video recording of the patient would be certainly more reliable” (Participant 115, attending physician).

Some participants expressed uncertainty about whether they would find a video helpful (n=6, 27%), offering that the utility of the video would depend on whether included elements of the neurological exam were directly relevant to a patient’s current ED presentation. They also expressed that they would find the video useful only if they were not able to obtain collateral information from a family member. One APP participant would not find a video useful and thought similar information could be obtained from other sources (n=1, 5%).

Key suggestions

Regarding both document and video options, participants emphasized that either would need to be brief and highlight high-yield information, given emergency clinicians’ time constraints. Participants noted that a potential benefit of either tool was saving clinicians’ time by having key information collated in one place, rather than necessitating that they “dig through the chart” (Participant 107, attending physician). Some suggested incorporation of a document or video into the EMR as a key report, similar to an advanced care planning tab. Participants recommended that reports or videos ought to be clearly dated and that content be periodically updated, given that changes in cognition occur over time, particularly among older adults with dementia. Further illustrative quotes for all themes are provided in Table 2.

**Table 2:** Illustrative Quotations for Selected Themes in a Qualitative Study of Emergency Clinicians’ Perceptions of Communication Tools to Establish the Mental Baseline of Older Adults

Code	Theme	Quotation
Establishing baseline mental status	Cognition: communication and memory	“What they can communicate? How’s their memory--if they remember short term things, if they remember long term things?” (Participant 111, attending physician) “Are they typically alert? Can tell you their name, date, year, where they are, all that stuff?” (Participant 123, physician assistant)
	Function: ADLs and mobility	“What their baseline is, again, is fundamental. What are they able to do, the ADLs [activities of daily living], what's their normal day like?” (Participant 105, nurse practitioner) “Do they have to use a walker, or do they climb stairs? Do they need a cane?” (Participant 109, resident physician)
Perceived utility of document or video	Potential to save clinician’s time	“This is information that isn't always included in a chart or it's buried in Epic and three thousand different notes, you know. So it's actually hard to get to.” (Participant 117, attending physician) “If I could see a video of what they're normally like for 30 seconds, I think that would eliminate a large fraction of what I'm trying to figure out from family. So it would be very helpful.” (Participant 118, resident physician)
	Potential to facilitate comparison of current presentation to reliable or objective information about patient’s baseline	“Our medical documentation is very, isn't that good. It's fine for like, extremities and heart and lungs, but it's not so good for neuro, especially mental status, as that's almost always barely mentioned, if at all.” (Participant 111, attending physician) “To the extent that the medical records can't be trusted, having an actual video recording of the patient would be certainly more reliable.” (Participant 115, attending physician) “I think a video would be super helpful…when it comes to patients that you're worried about stroke.” (Participant 109, resident physician)
	Potential to humanize or allow for more holistic view of a patient	“I think it just also adds to the patient encounter, because oftentimes in the emergency department, we don't have time to ask them these personal questions. So it's nice to learn more about the patient.” (Participant 125, physician assistant) “I think that would give you a fuller sense of…who the patient is.” (Participant 117, attending physician)
Key suggestions and concerns	Brevity	“I think in the emergency department, having about 10 percent of the [This is Me] document would be helpful…a briefer version of that for the emergency setting would be great.” (Participant 116, physician assistant) “Perhaps a more targeted or streamlined version of [This is Me] would be quite helpful.” (Participant 124, physician assistant) “I wonder how many people would review that [a video] in a time crunch situation.” (Participant 114, physician assistant) “That would be really helpful to [have] some kind of standardized question/answer, you know, quick--as quick as it could be to get all the elements.” (Participant 111, attending physician)
	Dating videos and keeping information up to date	“I think it has the potential to be helpful if it's recent and timely and hits on the major points that you would need to be aware of, such as a memory or cognition.” (Participant 117, attending physician) “My only worry would be that if somebody is in a subacute decline and the video is like a couple of months old…[If] that video was old and our deficit is small, that's not as big a deal, whereas if I'm deciding whether or not to give TPA to this person--and right now they can't form words fully, but it seems like a week ago they could, based on this video--like that would be very important.” (Participant 109, resident physician)

## Discussion

In this qualitative study, the majority of emergency clinicians viewed documents and videos depicting a patient’s baseline mental status as useful adjuncts in evaluating older adults with altered mental status. The development and use of document- or video-based communication tools could have important implications for improving the detection of delirium, which goes undetected in at least two-thirds of ED cases [7,9]. To our knowledge, pre-recorded video of a patient has not previously been used to help clinicians identify delirium or dementia; this novel and pioneering approach could be promising given the recent rapid expansion and increased acceptability of telemedicine and video-based care during the COVID-19 pandemic [22].

Participants’ emphasis on brevity as an essential feature of any communication tool reflects emergency clinicians’ time constraints and competing priorities when evaluating older adults [3,7,9]. In light of such challenges, the Geriatric Emergency Applied Research Network recommends that delirium detection measures should ideally not create significant time demands for emergency clinicians and nurses [7]. ED staff can perceive even brief geriatric screening exams that take less than one minute to perform as time-consuming and cognitively burdensome [23]. An important goal of a document or video would be saving clinicians’ time by collating key information in one place, which could be facilitated through EMR report functions, topical tabs, or information “dashboards.” A report in the EMR including key information about a patient’s baseline mental and functional status could be designed, highlighting a patient’s communication, memory, mobility, and ADLs, as prioritized by our participants. The Institute for Healthcare Improvement suggests a similar approach to incorporating the “4 Ms” of geriatrics (mentation, medications, mobility, what matters) into an EMR dashboard [24], and this strategy has been used to support interdisciplinary care for hospitalized older adults [25]. As interviewees also recognized, emergency and admitting teams do not often accurately document mental and cognitive status over - 60% of the time, as one study found [26] - making chart review a potentially unreliable source of information [1,10]. EMR dashboards have previously been shown to improve the accuracy of information conveyed and reduce medical errors in care handoffs [27]. Clear and succinct information about mental and functional status, whether in video or document format, could potentially help clinicians interpret and clarify information from other sources. Prior studies have shown that video messages can help emergency physicians and emergency medical services personnel clarify content in living wills and Physician Order of Life-Sustaining Treatment forms [17,18].

If document- or video-based interventions are developed to improve mental status evaluations of patients in the ED and within health systems more broadly, two topics raised by study participants merit further consideration. First, prioritizing content, specifically the key elements of a neurological or mental status examination that affect clinicians’ decision-making, will be important. Expert working groups from national geriatric emergency medicine associations could help determine the utility of capturing tests of baseline attention, concentration, or a brief delirium triage screen on video. Special attention should be paid to the standardization of prioritized content, as variety in the structure of such tools could introduce cognitive biases that affect decision-making. Second, stakeholders including ED administrators, end-users, and information and technology services teams should inform the accessibility of document- or video-based tools. Incorporation of video or document adjuncts into the EMR could address participants’ suggestions that information must be clearly dated and updated to allow robust comparison with a patient’s ED presentation. End-users and geriatrics experts should be queried about ideal time points for updating communication tools, such as after a care transition and recovery following hospitalization. Given potential concerns over video storage and retrieval, a smartphone-accessible online platform represents an alternative feasible system [17,18,28]. One such system called My Informed Decision on Video (MIDEO), designed to communicate older adults’ advanced care planning wishes, utilizes identification cards with QR codes allowing video retrieval in 10 seconds or less [28]. As MIDEO is external to the EMR, it is accessible across health systems and EMRs, and patients and caregivers can update video messages as their care priorities change [28]. Overall, future work should focus on how to make communication tools standardized, consistent, up to date, and relevant to ED clinical decision-making. Additional considerations which did not emerge from this study, but are nonetheless important, include but are not limited to patient consent and involvement in the generation of materials.

Limitations

This study relied on a convenience sample of volunteer participants from two urban academic hospitals and one academic-affiliated community hospital in the Northeast. Interviewees may have had a special interest in this topic and may not be representative of emergency clinicians within their institutions. Findings may not capture perspectives of emergency clinicians in rural community settings or other regions of the United States. We performed our study during the COVID-19 pandemic, and increased challenges in evaluating older adults during this time could have affected participants’ interest in and perceived utility of communication tools. Additionally, while the strength of qualitative research is eliciting participants’ perceptions and values about a topic, a larger-scale survey could provide information about the broader acceptability of communication tools and represents an important direction for future research. This study focused on emergency clinicians’ perceptions of novel communication tools that have not been validated in clinical settings. Additionally, the proposed tools would require health system resources for generation, updating, and maintenance involving multiple other stakeholders such as primary care clinicians and information technologists, whose perspectives were not sought for this study and represent an area of future inquiry.

## Conclusions

Evaluating older adults with changes in mental status in the ED poses challenges to clinicians, who must obtain collateral information from various sources to establish a patient’s mental baseline and understand deviation from it. In our study, the majority of emergency clinicians interviewed perceived utility in a document or video depicting an older adult’s baseline mental status. Such adjuncts could improve emergency clinicians’ evaluations of geriatric patients while potentially enhancing their ability to detect delirium.
